# Post-Modern Medicine: A Framework for Integrating Traditional and Modern Healthcare: Post-modern Medicine

**DOI:** 10.31661/gmj.v14i.3791

**Published:** 2025-09-12

**Authors:** Hassan Akbari

**Affiliations:** ^1^ Department of Pathology, Shahid Beheshti University of Medical Sciences, Tehran, Iran

**Keywords:** Post-modern Medicine, Traditional, Modern Healthcare

## Abstract

Post-modern medicine signifies a paradigm shift in healthcare by integrating the strengths of both modern biomedical science and traditional medical systems. This article explores the potential of a holistic approach that combines evidence-based practices with time-tested methodologies rooted in historical medical traditions. Emphasis is placed on prevention, diagnosis, and treatment to achieve a more comprehensive and personalized understanding of health and disease. By examining the unique contributions of each system, this integrative perspective aims to enhance patient outcomes in both human and veterinary medicine.

## Introduction

Over the centuries, healthcare has undergone substantial transformations driven by advances in scientific knowledge and shaped by diverse cultural and philosophical traditions. Modern medicine has emerged as a dominant system characterized by its technical precision, pharmacological innovations, and commitment to evidence-based practices. In parallel, traditional medicine systems—including but not limited to Traditional Chinese Medicine (TCM), Ayurveda, and Iranian Medicine—have offered holistic frameworks centered on prevention, lifestyle modifications, and individualized care [1][2].

The emerging concept of post-modern medicine represents a paradigm shift that transcends the limitations of both paradigms. Rather than merely juxtaposing conventional and alternative therapies—as often seen in integrative medicine—post-modern medicine seeks to synthesize the strengths of modern science with the philosophical depth and preventive orientation of traditional systems [3]. This hybrid model emphasizes patient-centered care, cultural contextualization, and a multidimensional understanding of health and disease.As chronic and lifestyle-related conditions increasingly challenge healthcare systems worldwide, a more inclusive, interdisciplinary medical model is needed. Post-modern medicine aims to bridge the gap between mechanistic and holistic approaches, fostering a healthcare paradigm that is both scientifically rigorous and culturally adaptable [4][5][6].

## The Capacities of Modern Medicine

Modern medicine is founded on a rigorous scientific framework that prioritizes accuracy, reproducibility, and technological advancement. It offers numerous strengths that have transformed the diagnosis and management of diseases:

1. Technological Innovation: Modern diagnostic modalities—such as MRI, CT scans, PET imaging, and next-generation sequencing—provide highly detailed insights into anatomical structures and disease mechanisms.

2. Pharmacological Developments: A continuously expanding pharmacopeia enables targeted interventions for a wide range of acute and chronic conditions, often grounded in randomized controlled trials.

3. Evidence-based Practices: Clinical decision-making is guided by systematic reviews, meta-analyses, and evidence hierarchies, ensuring interventions are both safe and effective according to current scientific standards.

4. Emergency and Critical Care: Rapid-response systems, including trauma care units, intensive care units (ICUs), and emergency surgery protocols, are optimized to manage life-threatening conditions with high efficiency and precision [7].

## Traditional Medicine

### Strengths and Scientific Validation

Traditional medicine encompasses diverse systems, including Ayurveda, Traditional Chinese Medicine (TCM), Unani, Iranian medicine, and Indigenous practices [8]. Key strengths include:

1. Holistic Approach: Emphasis on the interconnectedness and balance between mind, body, and environment.

2. Preventive Care: Focus on proactive lifestyle interventions and natural remedies to reduce the risk of illness.

3. Personalized Treatment: Consideration of individual constitutions, temperaments, and unique health needs.

4. Natural Therapies: Utilization of plant-based medicine, manual therapies such as acupuncture, and other non-invasive modalities.

Recent evidence from clinical trials and meta-analyses has established a growing scientific basis for the efficacy of traditional medical practices. For instance, a systematic review demonstrated that acupuncture significantly alleviates chronic pain conditions [9][10], while a meta-analysis confirmed the efficacy of Ayurvedic interventions in managing type 2 diabetes [11].

## WHO Traditional Medicine Strategy (2014–2023)

In response to the widespread global use of traditional and complementary medicine (T&CM), the World Health Organization (WHO) launched its Traditional Medicine Strategy 2014-2023 with the aim of integrating safe and effective traditional practices into national health systems. The strategy sought to promote the responsible use of traditional medicine while ensuring patient safety, enhancing regulatory frameworks, and encouraging evidence-based approaches [12].

### Main Objectives of the Strategy

The overarching goal of the WHO Traditional Medicine Strategy was to support Member States in developing proactive policies and regulatory measures to strengthen the role of traditional medicine in achieving universal health coverage (UHC). The strategy emphasized three strategic objectives:

• To build the knowledge base and formulate national policies.

• To strengthen safety, quality, and effectiveness through regulation and standardization.

• To promote universal health services by integrating T&CM into health systems where appropriate.

These objectives were aligned with global health priorities, acknowledging the contribution of T&CM in the prevention and management of both communicable and non-communicable diseases [12].

### Key Strategic Areas

To realize its objectives, the strategy highlighted several areas of action:

A. Research and Evidence Generation: WHO underscored the need for rigorous scientific evaluation of traditional medicines to ensure their safety, efficacy, and quality. Encouraging clinical trials, pharmacological studies, and systematic reviews were considered essential to inform policy and clinical practice [13].

B. Development of National Policies and Regulations: The strategy encouraged Member States to develop comprehensive national frameworks for the regulation, licensing, and monitoring of traditional medicine practitioners and products. It advocated for harmonization of standards, especially for herbal medicines, to safeguard public health.

C. Education and Capacity Building: Training programs were recommended to equip healthcare professionals and T&CM practitioners with the necessary competencies. WHO emphasized the need for standardized curricula and professional accreditation to ensure consistent service delivery.

D. Health Promotion and Disease Prevention: Recognizing the preventive potential of traditional medicine, the strategy encouraged its integration into national health promotion campaigns. Practices such as herbal therapy, acupuncture, and dietary interventions were promoted to complement biomedical care [12].

### Challenges and Barriers

Despite its ambitious goals, the implementation of the WHO strategy faced several barriers. These included limited scientific evidence supporting certain T&CM interventions, insufficient regulatory oversight in many countries, and skepticism from the conventional medical community. Additionally, disparities in access and the risk of misuse of traditional remedies posed significant concerns [14].

### Future Prospects

The WHO Traditional Medicine Strategy (2014-2023) served as a catalyst for the global recognition and responsible integration of T&CM. Several countries have since taken steps to institutionalize traditional medicine within their health systems. As WHO prepares for future updates beyond 2023, the emphasis is likely to shift further toward evidence-based practice, international collaboration, and digital health solutions to support traditional medicine [15].

## Improvement of Clinical Outcomes Using Both Traditional and Modern Medicine

**Figure-1:**
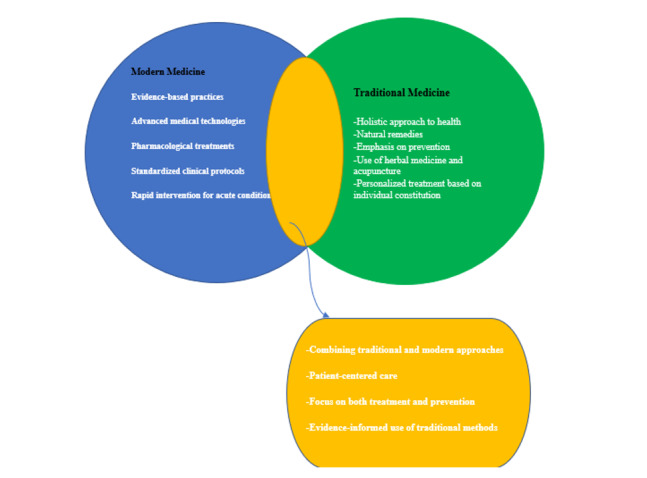
Figure 1. This diagram illustrates the distinctive features and areas of overlap between modern and traditional medical systems. On the left, modern medicine is characterized by evidence-based practices, cutting-edge diagnostic tools, and pharmacological interventions. On the right, traditional medicine emphasizes holistic philosophies, the use of natural remedies, and a strong focus on preventive care. In the center, the intersection highlights integrative healthcare models that incorporate elements from both paradigms to enhance patient outcomes.

In recent years, growing evidence has shown that combining traditional medicine with modern treatments can enhance clinical outcomes (Figure-1), particularly in areas such as oncology, chronic disease management, and pain relief. For example, a 2022 clinical study demonstrated that integrating Traditional Chinese Medicine (TCM) with chemotherapy significantly improved the quality of life for breast cancer patients by reducing fatigue, nausea, and psychological stress associated with treatment. This integrative approach has also proven beneficial for patients who either fail to respond adequately to conventional therapies or experience severe side effects. Another investigation involving cancer patients reported that the use of traditional therapies alongside standard chemotherapy protocols led to better pain control and overall symptom management compared to patients receiving modern treatments alone [16][17].

### Avicenna’s Description of Pulse Diagnosis

In his seminal medical encyclopedia, The Canon of Medicine, Avicenna (Ibn Sina) provided an extensive and systematic account of pulse diagnosis, a central element in traditional Greco-Arabic medicine. He meticulously classified different types of pulses based on their rhythm, speed, strength, volume, and regularity, offering insights that were remarkably sophisticated for his era. Pulse diagnosis in Avicenna’s framework served not only as a diagnostic tool for detecting disease but also as a means of assessing emotional and psychological states. He described more than ten distinct types of pulses, each associated with particular pathological or physiological conditions. For instance, a "serpent-like" pulse could indicate severe fever or weakness, whereas an "ant-like" pulse might reflect chronic conditions or old age [18].

Avicenna emphasized the importance of environmental and individual factors—such as age, temperament (mizaj), diet, and emotional status—in interpreting pulse variations. His comprehensive analysis demonstrates a fusion of observational precision and theoretical knowledge that significantly influenced both Islamic and European medicine until the modern era.

Contemporary historians regard Avicenna’s work on pulse diagnosis as a pivotal contribution to the advancement of pre-modern medical diagnostics, illustrating the depth and rationality of traditional Islamic medical sciences [19].

## The Impact of Complementary and Integrative Medicine on the Quality of Life in Cancer Patients

Complementary and integrative medicine (CIM) has gained considerable attention in oncology due to its potential to enhance quality of life (QoL), alleviate symptoms, and improve emotional well-being in cancer patients. CIM encompasses a variety of practices, including acupuncture, mind-body therapies, herbal medicine, and nutritional interventions, which are used alongside conventional cancer treatments [20].

### Enhancing Quality of Life Through Symptom Management

Cancer patients often endure a wide range of physical and psychological symptoms such as fatigue, pain, nausea, anxiety, and depression, particularly during chemotherapy and radiotherapy. Studies have shown that CIM interventions—particularly acupuncture, yoga, and mindfulness-based stress reduction (MBSR)—can effectively mitigate these symptoms and improve patients' overall well-being [21].

For instance, a randomized controlled trial demonstrated that acupuncture significantly reduced chemotherapy-induced nausea and vomiting in breast cancer patients [22]. Similarly, mind-body practices like meditation and guided imagery have been associated with reduced anxiety and better sleep quality in cancer survivors [23].

### Psychosocial Benefits and Emotional Support

CIM also offers psychosocial benefits by fostering a sense of control, resilience, and hope. Integrative approaches provide patients with tools to cope with the emotional burden of cancer, often empowering them to play a more active role in their care. Group-based interventions such as art therapy, tai chi, and support circles have been shown to improve mood and reduce distress levels among cancer patients [24].

### Safety and Integration into Oncology Care

While CIM has shown promise, its integration into clinical oncology must be approached cautiously. Evidence-based evaluation, practitioner training, and patient safety are critical. According to the Society for Integrative Oncology (SIO), oncologists should engage in shared decision-making with patients and guide them toward safe and effective CIM modalities [25].

Furthermore, institutional adoption of integrative oncology programs has been associated with higher patient satisfaction and improved treatment adherence, particularly in centers that offer multidisciplinary support and individualized care plans [26].

### Current Evidence and Research Gaps

Despite growing evidence supporting CIM in cancer care, limitations remain. Many studies lack rigorous methodology, standardized outcome measures, or long-term follow-up. More high-quality randomized trials and meta-analyses are needed to validate specific therapies and define their role in various cancer types and stages.

### Integration of Traditional Chinese and Western Medicine in COVID-19 Treatment

The integration of Traditional Chinese Medicine (TCM) with Western medical approaches has demonstrated promising outcomes in the management of COVID-19. A 2022 systematic review and meta-analysis by Zhang et al. highlighted that combining TCM with conventional therapies not only alleviated the severity of symptoms but also improved clinical outcomes such as reduced duration of hospitalization and enhanced recovery rates. The study emphasized the role of specific TCM formulations—such as Lianhua Qingwen, Jinhua Qinggan, and Qingfei Paidu decoctions—in modulating inflammatory responses, boosting immune function, and mitigating pulmonary complications associated with SARS-CoV-2 infection. These herbal interventions, when used alongside antiviral and supportive treatments, contributed to better prognosis and lower progression to severe disease stages. This integrative model reflects the broader potential of complementary medicine in pandemic contexts, provided that its use is evidence-based and guided by rigorous clinical evaluation. The findings support the continued investigation of traditional practices as adjuncts to modern treatment protocols, especially in respiratory infections with high morbidity [27]. While historical and contemporary examples illustrate the potential benefits of traditional and integrative approaches, it is equally important to critically assess the complexities and limitations involved in merging diverse medical paradigms.

### The Challenges of Integration and The Necessity of A Critical Approach

Integrating modern and traditional medicine requires careful navigation of their fundamental differences, particularly regarding diagnostic criteria, treatment standards, and the hierarchy of scientific evidence. While traditional systems offer valuable experiential knowledge, their principles often lack empirical validation through randomized controlled trials (RCTs) and meta-analyses, which are the cornerstones of modern clinical decision-making. This discrepancy raises a critical concern: could integration lead to contradictions in care? For instance, patients might forego proven treatments such as chemotherapy in favor of unproven herbal remedies, potentially compromising outcomes. Furthermore, integration without regulatory oversight may exacerbate healthcare inequities. Wealthier populations might access integrated services as a complement to high-quality biomedical care, while marginalized groups may receive only underfunded traditional treatments. This dual-track system risks deepening disparities rather than improving access. Thus, the path forward should not be uncritical integration, but rather selective incorporation of traditional practices that demonstrate safety and efficacy through rigorous scientific testing [28]. Instead of merging frameworks, the focus should be on using traditional medicine as a resource for potential drug discovery and supportive care, with proper clinical validation.

## The Legacy of Iranian Medicine

Iranian medicine, which has evolved over centuries and is deeply rooted in the scholarly works of figures such as Avicenna (Ibn Sina) [29], offers a distinct framework for understanding health and disease. Its notable characteristics include:

1. Comprehensive Health Philosophy: An integrative view that encompasses physical, psychological, and spiritual dimensions of health.

2. Dietary Therapy: Emphasis on the preventive and therapeutic role of diet, as extensively discussed in Avicenna’s "Canon of Medicine".

3. Natural Remedies: Therapeutic use of herbs, minerals, and lifestyle interventions as primary tools for maintaining and restoring health.

4. Historical Contributions: Significant influence on the development of modern disciplines such as pharmacology, anatomy, and clinical diagnostics.

## Concepts of Traditional Medicine Scientists

The foundational concepts introduced by historical figures in traditional medicine have significantly influenced modern integrative healthcare systems. These pioneers emphasized the interplay between physical, mental, and environmental factors in disease prevention and treatment.

1. Avicenna (Ibn Sina): Author of The Canon of Medicine, Avicenna advocated for the equilibrium of bodily humors (blood, phlegm, yellow bile, and black bile) as the basis for health. His holistic perspective incorporated mental health as a critical component of overall well-being, aligning with contemporary notions of homeostasis and metabolic balance. Modern integrative medicine continues to validate his emphasis on internal balance as essential to maintaining health [30][31][32].

2. Hippocrates: Regarded as the "Father of Medicine," Hippocrates introduced the Hippocratic Oath, which remains foundational to medical ethics. He promoted healing through natural means such as nutrition, exercise, and lifestyle modifications. Current research supports his principles, emphasizing lifestyle medicine and the preventive role of diet and physical activity in reducing the burden of chronic diseases [33].

3. Zhang Zhongjing (Traditional Chinese Medicine - TCM): Author of Shang Han Lun, Zhang emphasized pattern-based diagnosis and personalized herbal prescriptions. These approaches are paralleled in modern psychosomatic medicine, which acknowledges the impact of mental and emotional states on physical health. Recent studies confirm the relevance of TCM’s mind-body framework in contemporary holistic care [34].

4. Rhazes (Al-Razi): A prominent Persian scholar, Rhazes contributed extensively to clinical medicine through systematic observations and detailed case documentation. In his work Kitab al-Judari wa al-Hasbah, he was the first to clinically differentiate smallpox from measles, a breakthrough in infectious disease management [35].

5. Paracelsus: A European physician who blended alchemical principles with medical practice, Paracelsus introduced the Doctrine of Signatures, positing that the shape and color of plants hint at their therapeutic uses. His approach foreshadowed modern phytotherapy and the symbolic interpretation of natural remedies [36].

6. Charaka (Ayurveda): Compiler of the Charaka Samhita, Charaka emphasized digestion, ethical medical practice, and the balance of the three doshas (Vata, Pitta, and Kapha) as key to health. His framework closely aligns with preventive strategies in modern personalized and lifestyle medicine [37].

## World Health Organization’s Role in Integrative Medicine

The World Health Organization (WHO) has played a significant role in the global recognition and incorporation of traditional medicine into healthcare systems. Key aspects of the WHO's involvement include:

1. Traditional Medicine Strategy 2014-2023: This strategy provides a framework to integrate traditional medicine into health systems worldwide, aiming to strengthen healthcare delivery through diverse medical practices.

2. Universal Health Coverage: The WHO advocates for healthcare that is accessible, affordable, and culturally appropriate, including the integration of traditional healing practices where suitable.

3. Safety and Efficacy: The WHO encourages research to validate and standardize traditional remedies, ensuring that these treatments meet safety and efficacy standards comparable to modern medicine.

4. Global Collaboration: By fostering international partnerships, the WHO aims to combine the strengths of modern and traditional medicine, creating a more inclusive healthcare model.

## Integrating Modern and Traditional Medicine

The integration of modern and traditional medicine requires systematic and multidisciplinary efforts. This process involves:

1. Collaborative Research: Conducting rigorous studies to evaluate the efficacy of traditional remedies alongside conventional treatments is essential for developing an integrated healthcare model.

2. Education and Training: Healthcare providers must be equipped with knowledge of both modern and traditional systems to provide comprehensive, patient-centered care.

3. Policy Support: Governments and health authorities must develop regulatory frameworks that facilitate the recognition and integration of traditional practices within formal healthcare systems.

4. Patient-Centered Care: Empowering patients to make informed decisions about their treatment options, based on their values, is critical in an integrated healthcare system.

## Concrete Examples of Integrated Medicine

Several successful examples demonstrate the potential benefits of combining modern and traditional approaches:

1. Ayurvedic Hospitals in India: In places such as Kerala, Ayurvedic hospitals integrate modern diagnostic methods with traditional Ayurvedic consultations. This holistic approach allows for a comprehensive view of a patient's health, combining advanced diagnostics with time-tested therapeutic practices [38].

2. Traditional Chinese Medicine in Cancer Care: Hospitals like Beijing University of Chinese Medicine use Traditional Chinese Medicine (TCM) alongside conventional cancer treatments like chemotherapy. Techniques such as acupuncture help mitigate side effects and enhance patient well-being during cancer treatment [39].

3. Acupuncture in Pain Management: In the United States, integrative pain management clinics combine acupuncture with conventional medical therapies to treat chronic pain, demonstrating the effectiveness of such an approach [40].

4. Global Integrative Approaches: In countries like Germany and Canada, frameworks for integrative medicine are already in place, supporting collaboration between conventional and traditional practitioners. For instance, the German Society for Integrative Medicine advocates for research, training, and policy-making in this area [41].

## Benefits of Integrating Modern and Traditional Medicine

The integration of these two systems offers multiple benefits:

1. Cultural Preservation: The integration of traditional medicine helps preserve cultural heritage and promotes the transfer of intergenerational knowledge. This process strengthens community identity and fosters respect for diverse medical traditions.

2. Economic Opportunities: The expansion of traditional medicine can stimulate local economies by creating jobs in cultivation, preparation, and distribution of natural remedies. Additionally, it supports indigenous communities by ensuring fair economic returns from the sustainable harvesting of medicinal plants.

3. Accessibility: Traditional medicine often relies on locally available resources, making healthcare more accessible in remote or underserved areas.

4. Sustainability: By emphasizing renewable and natural resources, traditional medicine can reduce the ecological impact of healthcare practices.

5. Improved Outcomes: The synergy between modern and traditional approaches can lead to more effective prevention and treatment strategies.

6. Empowerment: Integrating both systems encourage self-reliance in health management through lifestyle modifications and the use of accessible, natural remedies.

## Applications in Prevention

The integration of modern and traditional medicine has profound implications for preventive strategies.

Modern medical tools, such as screening methods, can identify health risks early, while traditional practices like dietary adjustments and stress management contribute to improved resilience. For instance, TCM’s focus on balancing Qi aligns with modern understanding of metabolic health, promoting overall well-being and preventing disease [42].

## Applications in Diagnosis

While modern diagnostic technologies offer unparalleled accuracy, traditional techniques like pulse diagnosis in Ayurveda and TCM provide additional insights into systemic imbalances. Combining these approaches can offer a more comprehensive view of a patient's health.

## Applications in Treatment

Integrated treatment strategies can optimize outcomes. For example:

• Cancer Care: The combination of chemotherapy with herbal supplements may help reduce side effects and improve patient quality of life.

• Chronic Diseases: Modern insulin therapy for diabetes can be complemented by dietary changes and herbal treatments, improving long-term management.

• Pain Management: Integrating conventional pharmaceutical treatments with acupuncture or mindfulness practices can provide holistic pain relief.

## Challenges and Ethical Considerations

The integration of traditional and modern medicine faces several challenges, including:

1. Scientific Validation: Traditional medicine must undergo rigorous scientific validation through randomized controlled trials (RCTs) to establish safety and efficacy. This is critical for gaining acceptance within the global medical community.

2. Ethical Concerns of Commercialization and Cultural Appropriation: As traditional medicine becomes more popular globally, concerns regarding commercialization and cultural appropriation arise. It is essential to respect the cultural origins of traditional practices and ensure that indigenous community’s benefit from the use of their knowledge, including recognizing intellectual property rights and providing fair compensation to traditional healers.

3. Safety Risks of Herbal Medicine Interactions: Herbal remedies, while beneficial, can interact with pharmaceuticals, potentially causing adverse effects or reducing the efficacy of conventional treatments. Regulatory agencies like the U.S. Food and Drug Administration (FDA) and the European Medicines Agency (EMA) are responsible for ensuring the safety of herbal products by establishing labeling guidelines and monitoring adverse reactions.

4. Access and Equity: Ensuring equitable access to integrated medical services is crucial to promote health equity, especially in low-income and rural areas.

## Conclusion

In conclusion, the integration of traditional and modern medicine presents both opportunities and challenges in developing a more comprehensive healthcare system. While combining the evidence-based approaches of modern medicine with the holistic and preventive aspects of traditional practices holds promise, it is essential to carefully evaluate the safety, efficacy, and cultural implications of such integration. This process must be grounded in rigorous empirical research to avoid contradictions and ensure patient safety. By promoting mutual understanding and respect between medical systems, we can improve healthcare outcomes, particularly in diverse cultural settings. However, we must be cautious not to exacerbate health inequalities, ensuring that both systems are accessible to all. In times of global health crises, such as pandemics or the increasing burden of chronic diseases, an evidence-based integrated approach can offer innovative and culturally sensitive solutions. Ultimately, the promotion of integrative medicine should prioritize empirical validation and the protection of patient safety while fostering better healthcare outcomes for individuals and communities worldwide.

## Conflict of Interest

There is no conflict of interest.
